# Practical Particulate Matter Sensing and Accurate Calibration System Using Low-Cost Commercial Sensors

**DOI:** 10.3390/s21186162

**Published:** 2021-09-14

**Authors:** Hyuntae Cho, Yunju Baek

**Affiliations:** 1 School of Digital Media Engineering, Tongmyong University, Busan 48520, Korea; 2 School of Computer Science and Engineering, Pusan National University, Busan 46241, Korea; yunju@pusan.ac.kr

**Keywords:** particulate matter, aerosol, micro dust, yellow dust, calibration, low-cost

## Abstract

Air pollution is a social problem, because the harmful suspended materials can cause diseases and deaths to humans. Specifically, particulate matters (PM), a form of air pollution, can contribute to cardiovascular morbidity and lung diseases. Nowadays, humans are exposed to PM pollution everywhere because it occurs in both indoor and outdoor environments. To purify or ventilate polluted air, one need to accurately monitor the ambient air quality. Therefore, this study proposed a practical particulate matter sensing and accurate calibration system using low-cost commercial sensors. The proposed system basically uses noisy and inaccurate PM sensors to measure the ambient air pollution. This paper mainly deals with three types of error caused in the light scattering method: short-term noise, part-to-part variation, and temperature and humidity interferences. We propose a simple short-term noise reduction method to correct measurement errors, an auto-fitting calibration for part-to-part repeatability to pinpoint the baseline of the signal that affects the performance of the system, and a temperature and humidity compensation method. This paper also contains the experiment setup and performance evaluation to prove the superiority of the proposed methods. Based on the evaluation of the performance of the proposed system, part-to-part repeatability was less than 2 μg/m^3^ and the standard deviation was approximately 1.1 μg/m^3^ in the air. When the proposed approaches are used for other optical sensors, it can result in better performance.

## 1. Introduction

According to a 2014 report by the WHO, 4.3 and 3.7 million people lose their lives due to indoor and outdoor air quality pollution, respectively [1,2]. Specifically, particulate air pollution such as yellow dust and fine dust is getting serious every year in many countries across Asia, the Middle East, Africa, and Europe. Such particulate air pollution is caused by automobiles, thermal power plants, combustion of coal, sand of the desert, etc., and may also occur when food is grilled or fried indoors. Recently, as the quality of life has improved in many countries, interest and knowledge about particulate air pollution such as particulates is increasing, and cutting-edge technologies and products such as air purifiers and air quality monitoring systems are being launched in the market. However, since most air quality monitoring systems and air purifier systems use low-cost sensors, the accuracy and performance of the sensors are relatively low [3,4,5,6,7].

Various methods such as gravimetric method, beta-ray-based measurement method, resonance frequency measurement method, and light scattering method for measuring particulates have been proposed [8,9,10,11,12,13,14,15]. In the case of gravimetric method [16], it is used as a standard measurement method because it has the best measurement accuracy. However, since it is expensive and bulky, it is not suitable for use in IoT devices or home appliances. Beta-ray absorption method (BAM) [17] collects particulates on a filter paper for a certain period of time, transmits beta-rays, and measures the concentration by the difference between the beta-rays absorbed and extinguished when the injected beta-ray passes through the particulates on the filter paper. This method has lower accuracy than the gravimetric method, but the price and size are relatively low. Tapered element oscillates microbalance (TEOM) [18] is a system that measures the amount of particulates in the air by detecting the change in resonance frequency according to the weight of the fine dust collected on the filter paper in the impactor. Lastly, the light scattering method irradiates a laser or IR LED, measures the intensity of light scattered by particulates, and converts it into the concentration of particulates. In systems such as IoT devices or air purifiers, low-cost light scattering methods are mainly used. However, the light scattering method is very vulnerable to external environments such as temperature, humidity, etc., and has accuracy and stability drawbacks, such as responding easily to the particle size and shape of particulates [19]. Some research to measure particulate matters in ambient environment have been performed [20,21,22,23]. We will discuss some of the studies that reported on the error factors and the calibration of the low-cost sensors in Section 2.

The aim of this study was to develop an accurate particulate matter sensing system using commercial coarse sensors. Most commercial PM sensors are inaccurate because they are based on the optical scattering method and are vulnerable to ambient environment change. First, this paper analyzed error factors in using the commercial PM sensor and designed the system to measure the concentration of ambient PM. Then, we proposed a PM calibration method with reduced noise, easy part-to-part repeatability compensation, and temperature and humidity compensation to correct measurement errors. In particular, the proposed method has an auto-fitting calibration for part-to-part repeatability to pinpoint the baseline of the signal that affects the performance of the system. As a result of the evaluation of the performance of the proposed system, part-to-part repeatability was less than 2 μg/m^3^ even though there is no special further calibration phase. This paper also conducted the evaluation for the power consumption of the system.

This paper is organized as follows. First, we present related work in Section 2 and then discuss a particulate matter sensing system with networking for household environment in Section 3. Section 4 describes the PM calibration method for high accuracy. In Section 5, we evaluate the performance, consisting of part-to-part repeatability, accuracy, and power consumption. Finally, Section 6 concludes this paper and proposes future work.

## 2. Related Work

Various studies have investigated the use of calibrations for low-cost sensors. Zaidan et al. [23] proposed a virtual sensor system based on machine learning. The proposed method collects a huge amount of accurate air quality data (PM2.5, CO_2_, etc.) from a reference instrument and then uses artificial intelligence to predict air quality from low-cost sensors in the real field. In their system, more accurate results can be obtained because machine learning is used to correct the difference between the reference instrument and low-cost sensors. However, for more accurate data, it is necessary to acquire a lot of data from the reference instrument and sensors, and since it is difficult to actually measure and learn it on-site, this is a drawback to their system.

Alfano et al. [8] reported a study on fine dust measurement. The study focused on classification and measurement methods by size of dust particles. They described the operating principles of the gravimetric method, tapered element oscillating microbalance (TEOM), beta ray attenuation (BAM), optical particle counters (OPC), and light scattering (especially Mie theory) as the measurement methods for particulate matter. They also described various laboratory-level chamber setups that can accurately measure particulate matter, and the need to compensate for temperature and humidity in order to improve the accuracy of the optical sensor. Their study is a good reference point for us because it majors on the accuracy of various PM sensors in the market.

Motlagh et al. [24] created a system that uses low-cost wearable sensors to obtain accurate information about public transport users being exposed to pollutants every day. In their study, the degree of exposure to pollutants was measured by measuring particulate matter. However, they only carried out experiments on the deviation between sensors using commercial sensors and reported nothing on sensor calibration and error factors.

Gressent et al. [25] conducted a large-scale project to view the distribution of air pollution in the entire city. Since the reference station network was sparse and expensive, many sensors (fixed low-cost sensors) were mounted on buildings and others (mobile low-cost sensor) mounted on moving vehicles to measure the concentration of particulate matter in the city center. They also used a large amount of observations provided by the sensors for urban-scale air quality mapping to show their potential added value with respect to the computation of dispersion models. Zaidan et al. [23] showed the accuracy of the low-cost sensor was improved while compensating for the difference with the reference instrument. Daily averages of estimated concentrations, reference observations, and hourly model outputs at each station were used to compare the performance of data fusion.

Miskell et al. [26] proposed simple, remote, and continuous calibration techniques for hierarchical networks, where well-maintained, high-accuracy instruments (proxy) and many low-cost sensors are deployed. The mean and standard deviation of the sensor data are matched with values derived from a proxy over the same time period to derive an estimate of the driving slope and offset.

Clements et al. [27] reported on a 2-day workshop where they discussed: (1) best practices for the deployment and calibration of low-cost sensor systems; (2) data standardization efforts and database design; (3) advancements in sensor calibration, data management, data analysis, and visualization; and (4) community panel (Lessons learned from research/community partnerships). The panel discussion summarized knowledge advances and project success, while highlighting the remaining questions, unresolved issues, and technical limitations in the realm of low-cost air quality sensors.

## 3. Particulate Matter Sensing and Calibration System

**Figure 1:**
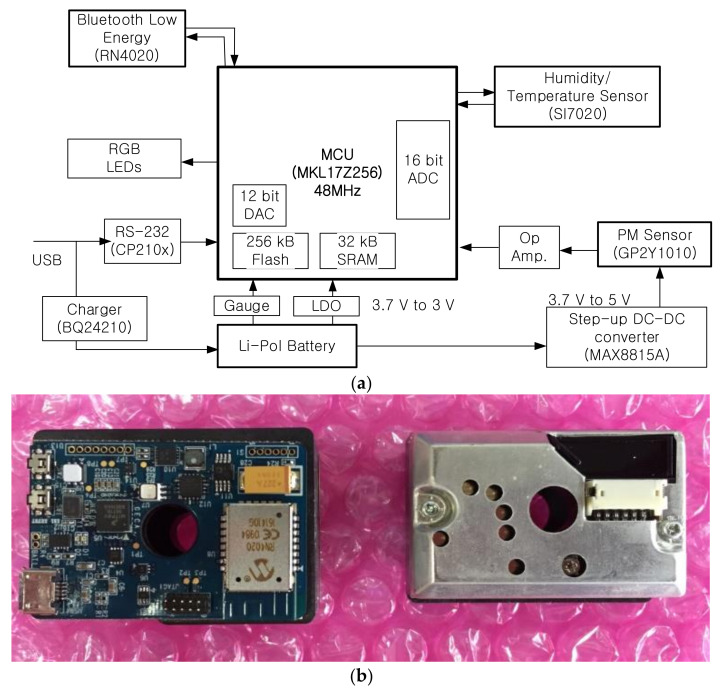
PM sensing system: (**a**) block diagram and (**b**) appearance.

Figure 1 contains a block diagram and the appearance of the PM sensing system, Table 1 shows the part list used in the system. The system is managed by NXP MKL 17 [28] (ARM Cortex-M0+) chip. This micro-controller unit (MCU) operates at 48 MHz clock and consumes 6.54 mA current at run mode and 3.31 uA at very-low-power stop mode. It also contains 256 KB flash and 32 KB SRAM memories. Moreover, it has 16-bits successive approximation (SAR) analog-to-digital converter (ADC) to provide high resolution in sensing PM. We employed Sharp GP2Y1010 PM sensor [29] for our low-cost commercial PM sensor to measure ambient particulate matters. GP2Y1010 operates at 5 V while other system components work at 3 V. Therefore, we used a step-up DC/DC converter to generate 5 V from the system operating voltage (3.0 V). The output of GP2Y1010 is inserted into the ADC channel of the MCU via operational amplifier (OP AMP). We also used a humidity and temperature sensor, Silicon labs SI7020 [30], to compensate for the error factors caused by humid and temperature. The sensed data is sent to remote smart devices via USB or Bluetooth low energy (BLE) [31]. Then, the remote smart device can get multiple data from multiple PM sensing systems. This function is one of the most important factors to measure air pollution i.e., houses with various rooms experience different environments, and therefore require multiple sensors for accurate sensing. Figure 1b shows the appearance of the PM sensing system we designed. It has a small form factor of 45 mm × 35 mm that is similar to the GP2Y1010 sensor. We designed the system for small form factor to use it in many applications.

## 4. Accurate Calibration Using Low-Cost Commercial Sensors

### 4.1. Operation Principle of the PM Sensor

**Figure 2:**
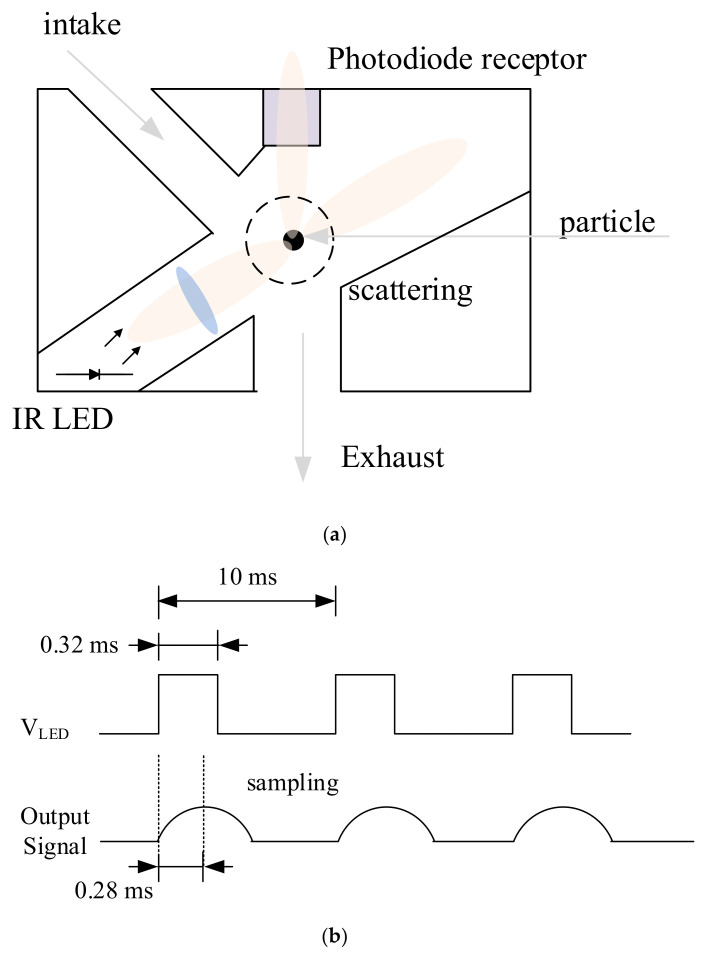
Output signal of the PM sensor: (**a**) operation principle and (**b**) input/output signals.

**Figure 3:**
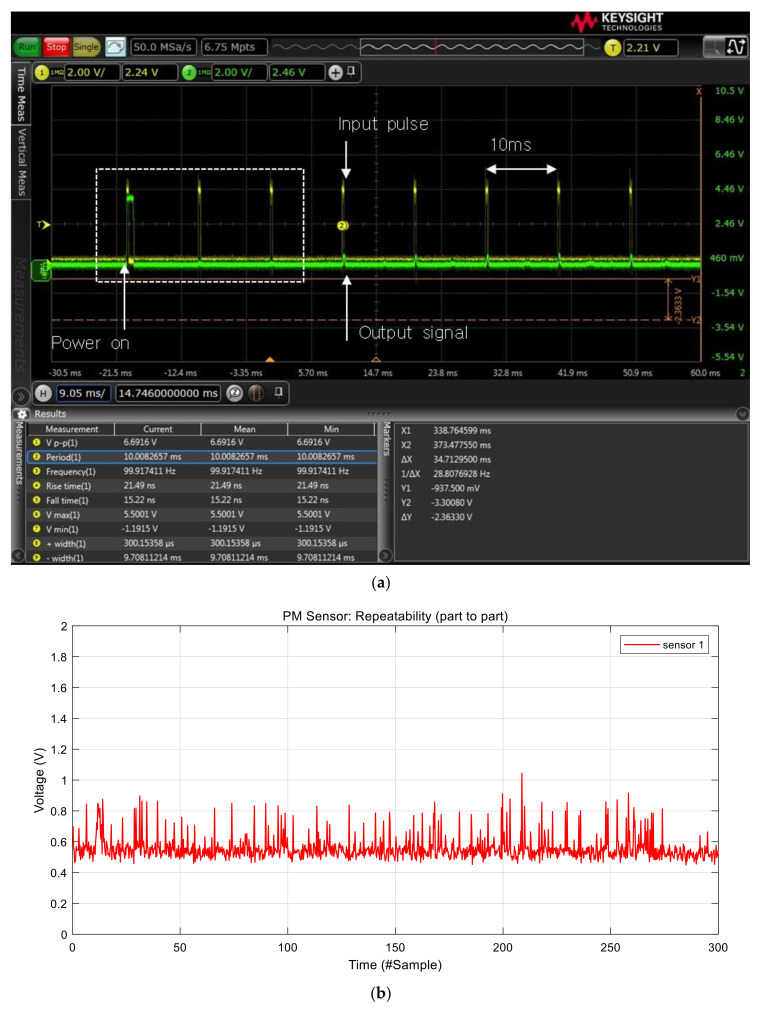
Output signal of the PM sensor: (**a**) oscilloscope capture and (**b**) the measured output data by the MCU.

In this system, we used Sharp GP2Y1010 (Japan) sensors for PM sensors to measure the concentration of particulate matters. Figure 2a illustrates the operation principle of the GP2Y1010. It employs light scattering as an operation principle: An infra-red (IR) light beam is emitted into a measurement chamber such that when dust is present, the light is refracted by particles and the amount of scattered light is detected by the internal photo diode. The GP2Y1010 uses pulse signals to emit IR light as shown in Figure 2b. The PM sensor basically requires consequent 100 Hz pulse signals to read output signals from the PM sensors. Each pulse signal has a pair of a high signal of 0.32 ms and a low signal of 9.68 ms. The PM sensor generates the highest output signals 0.28 ms after the start of the high signal, and then the output signal is converted into voltage and the concentration of particles through the ADC values. Figure 3 shows actual input pulse and output signals of the GP2Y1010 measured by an oscilloscope. The figure shows that it generates output signal after the forth input pulse from start. Therefore, the system discards the initial first three data.

**Figure 4:**
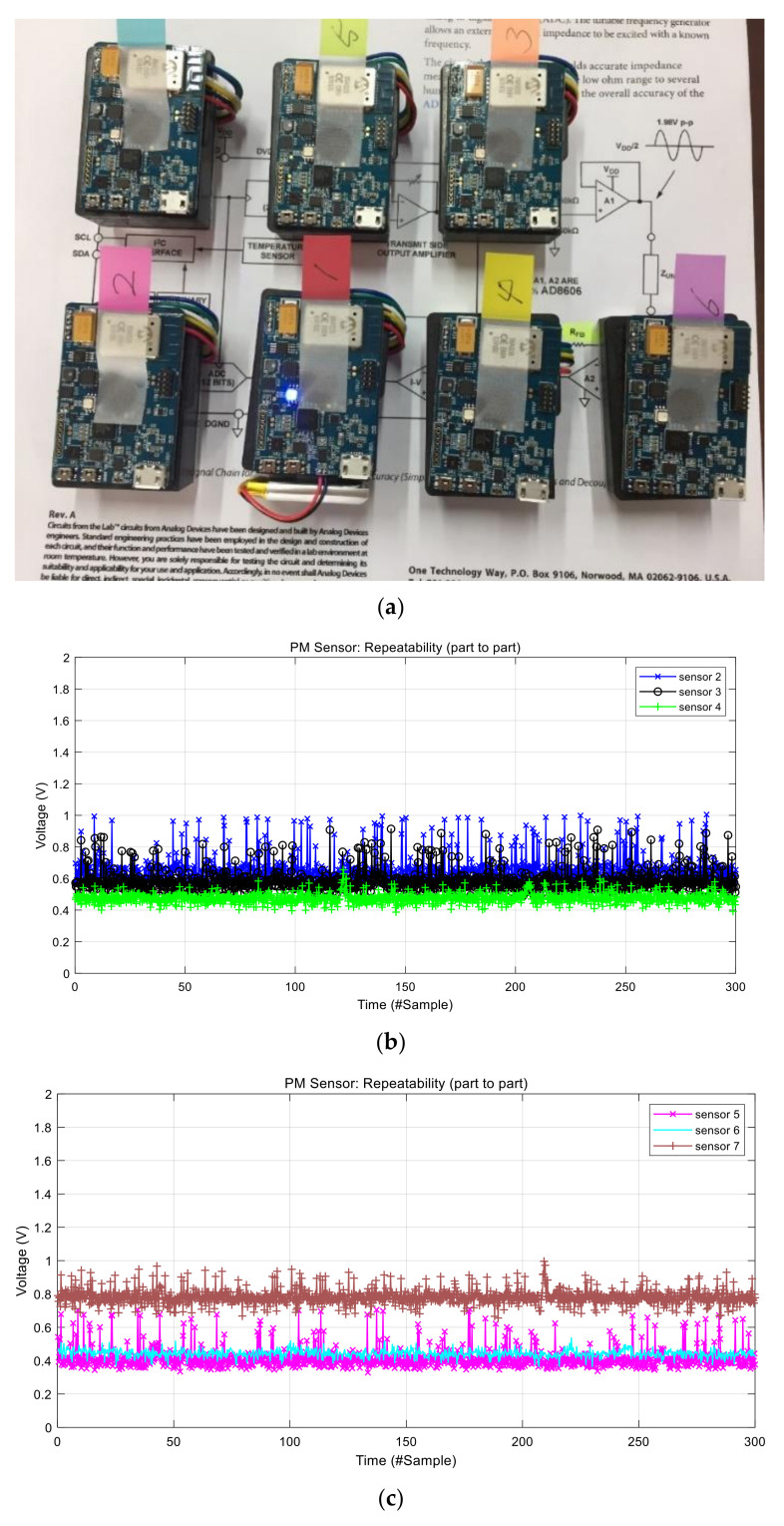
Experiment and output signals: (**a**) experiment environment, and (**b**,**c**) raw voltage data measured by the MCU.

We analyzed real output data of the GP2Y1010 sensor using seven sensors. The data were collected in clean air environment with no visible particles. In addition, we also blocked the air vent of the sensors as shown in Figure 4a. Figure 3b shows the raw output voltage signal of the PM sensor 1. The output signal has a lot of short-term noises with approximately 0.3 V variation. This short-term noise can result in reduced accuracy when measuring the concentration of particulate matters. Therefore, a short-term noise reduction approach is necessary. Figure 4b,c shows the raw output signals of the other sensors, indicating that each sensor has a short-term noise and part-to-part variation at the same time. The Figure implies that each sensor has a different baseline that is used as the intercept in the regression model to convert output voltages into the concentration of particulate matters. PM sensor 5 has the lowest baseline while PM sensor 7 has the highest baseline in Figure 3b and Figure 4b,c. The baseline difference among sensors should be eliminated to reduce part-to-part variation that decreases the measurement accuracy.

**Figure 5:**
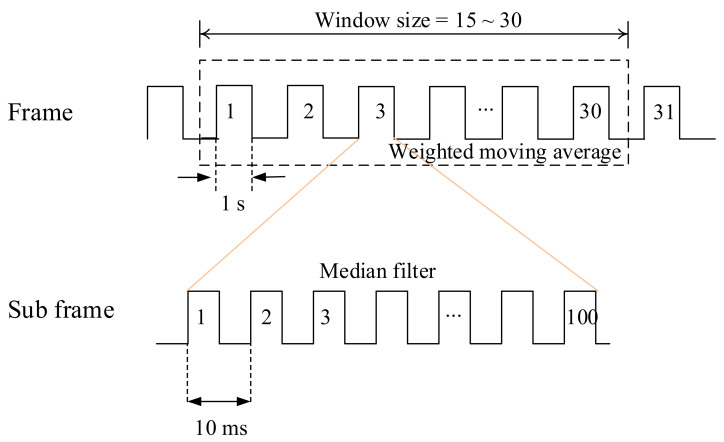
Sensing frame and sub frame.

To minimize the short-term noise and calibrate the baseline from part-to-part variation, we proposed a system that calculates the concentration of the PM every second. As shown in Figure 5, each sub frame has 100 sensing phases for 1 s and then the system calculates the median over the 100 samples. As the second phase is to further reduce the noise, we applied a weighted moving average filter with a window size of 30 s (i.e., 30 data points) on the data. Current output voltage, ${\overset{¯}{x}}_{i}$, is determined by Equation (1). This approach further reduces the noise of the data.

(1) $${\bar{x}}_{i}=\left(x_{i}\times \alpha \right)+\left(\frac{1}{n-1}\overset{i-1}{\underset{j=0}{\sum }}x_{j}\right)\times \left(1-\alpha \right)$$

where, *n* is the number of the window, *x* is the system input in discrete time domain, *n* is the time in discrete time domain, and α is the weight factor between the current measured value and other values in the moving window.

**Figure 6:**
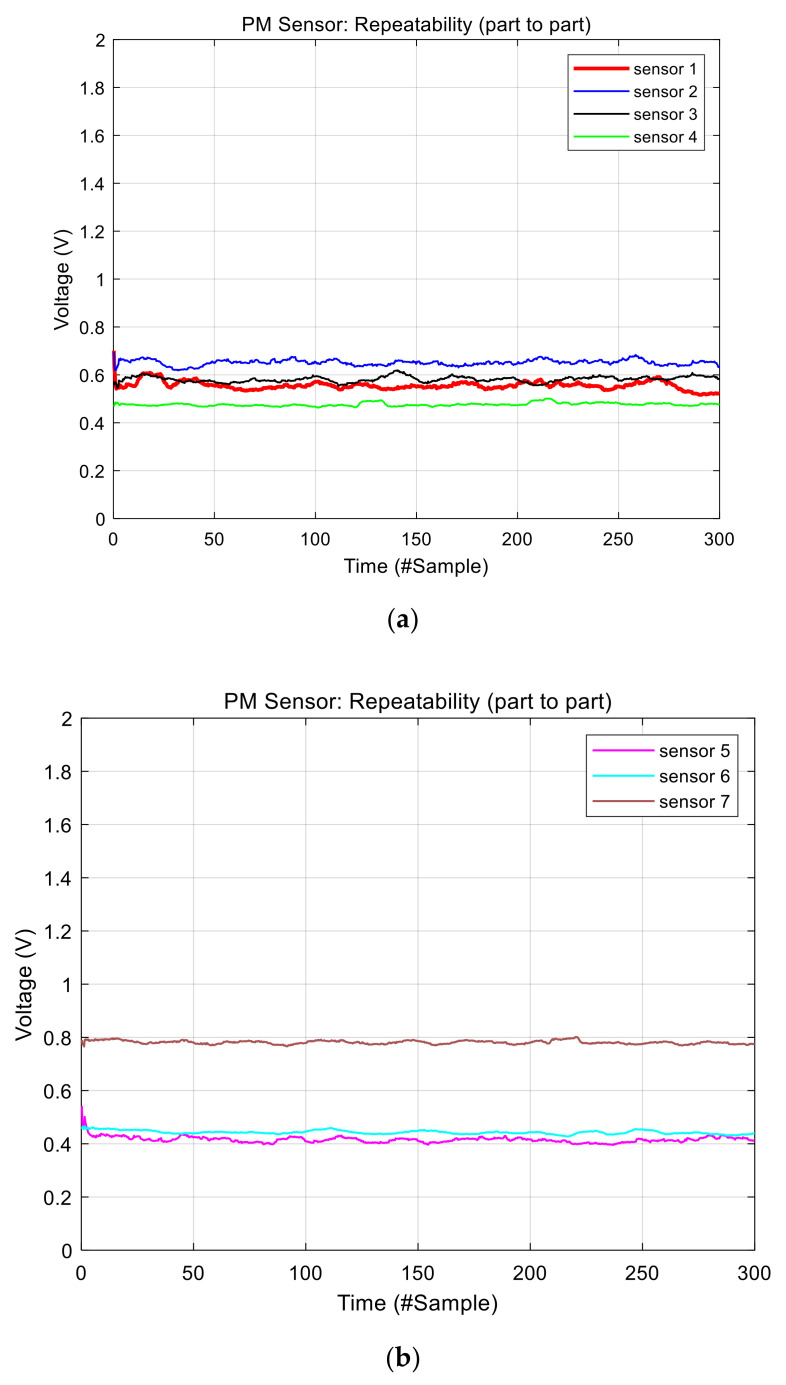
Result of the noise reduction: (**a**) data collected from sensor 1 to 4 and (**b**) data collected from sensor 5 to 7.

Then, we can convert output voltage into the concentration of the dust by using a regression model. The data sheet of GP2Y1010 shows an exemplary relationship between the dust and the sensor’s output voltage. Although we reduced noises, the sensors represent the different baseline voltages caused by part-to-part variation, which means they can have different intercepts in the linear function, as shown in Figure 6. As earlier mentioned, this affects the accuracy of the system sensing the concentration of dust particles. For instance, if we make a regression model based on sensor 4 and apply it to sensor 2 in Figure 6a, it will be judged that in sensor 2 there is quite a bit of dust particles even though there is no dust. So, all PM sensors should be calibrated by different baseline voltages or different intercepts.

**Figure 7:**
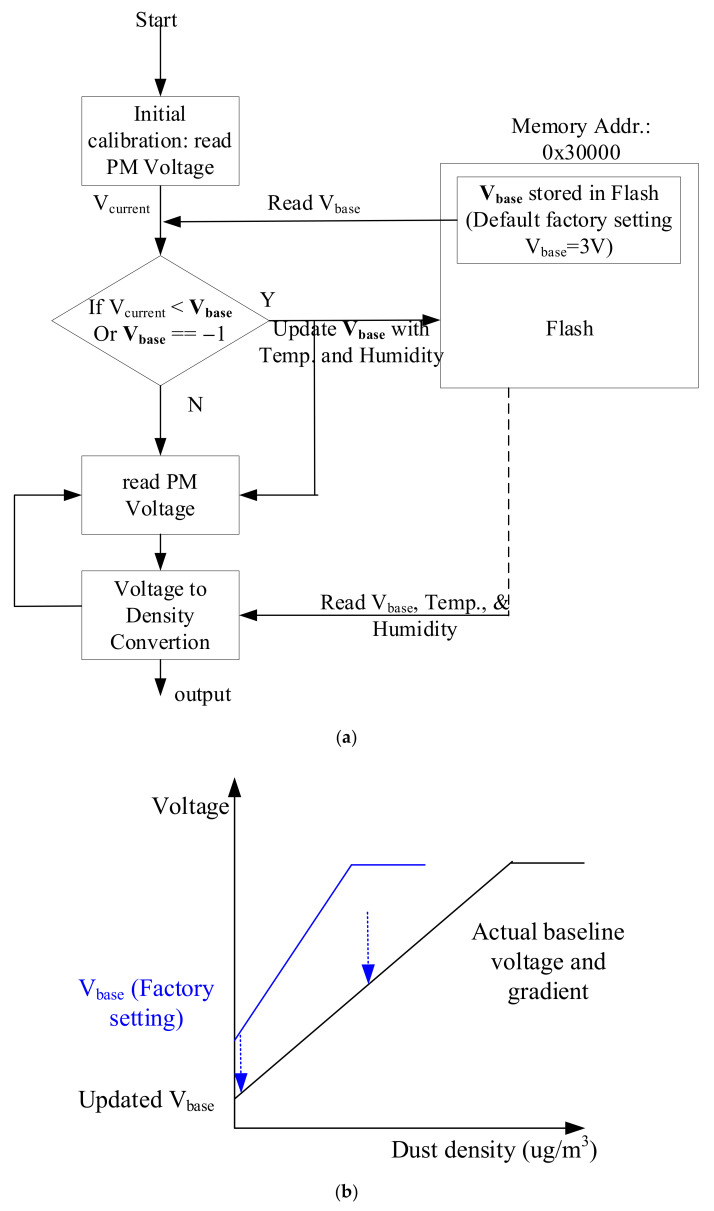
Baseline voltage calibration procedure: (**a**) procedure and (**b**) example.

This study proposed an autonomous baseline search procedure in the initial phase, as shown in Figure 7, to reduce calibration cost for mass production. After booing sequence, the system has an initial calibration phase where the system reads voltage values from the PM sensor and then compares the median value, *V*_current_, with a factory setting voltage value, *V*_base_, stored in non-volatile flash memory. If *V*_current_ is smaller than *V*_base_ or it is equal to ‘−1′, it updates *V*_base_ into flash memory. ‘−1′ means that it has never been calibrated and updated before. Figure 7b shows an example of *V*_base_ update. After finishing the initial calibration phase, it reads and calculates the concentration of particles using the information about baseline voltage. The converter between the voltages to the concentration reads the baseline (called the intercept) voltage from the internal flash memory that was updated during the initial calibration phases.

At this stage, one can calculate the concentration of particles and dusts using voltage values. The data sheet of GP2Y1010 shows an exemplary relationship between the dust density and the sensor’s output voltage in a simple linear function. We also got similar curves with the same gradient through experiments even though their baseline voltages were different, meaning that if we know the intercept, we can easily calculate the concentration of dust using the linear regression model. The concentration of dust is calculated by Equation (2)

(2) p(x)=a⋅x+b

where, *p*(*x*) is dust density, *a* is the gradient, *x* is the output voltage, and *b* is the *Y* axis intercept. The gradient, *a*, is gotten as approximately 0.17 by data sheet and experiments.

**Figure 8:**
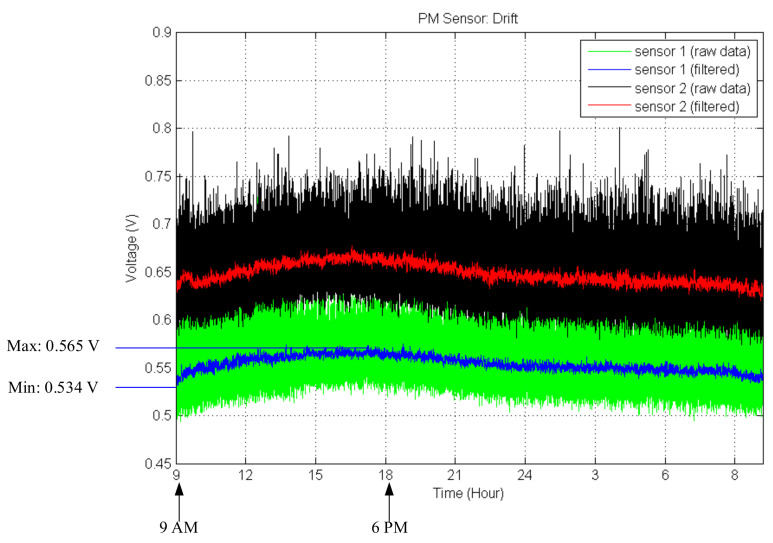
Drift experiment over time.

Matthias Budde et al. [32] introduced drift over time, and a separate calibration step for each sensor to determine its time-dependent drift factor *k*. Using this, they adjusted their calculation of *a* and *b*. Therefore, to verify them, we experimented and analyzed data for 24 h as shown in Figure 8. The graph seems to have drift for the initial 10 h. In the case of sensor 1, there is 11 milli-voltage difference (around 2 μg/m^3^). However, the sensors recovered after around 24 h. We found out that this drift is caused by temperature difference.

**Figure 9:**
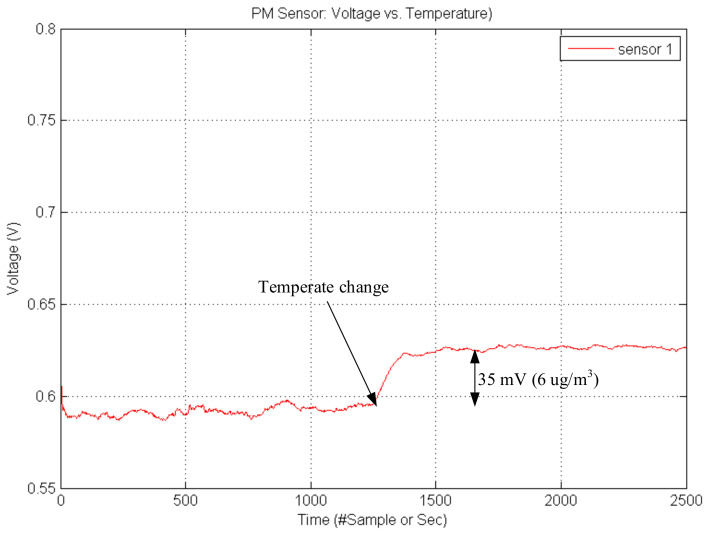
Temperature affection.

**Figure 10:**
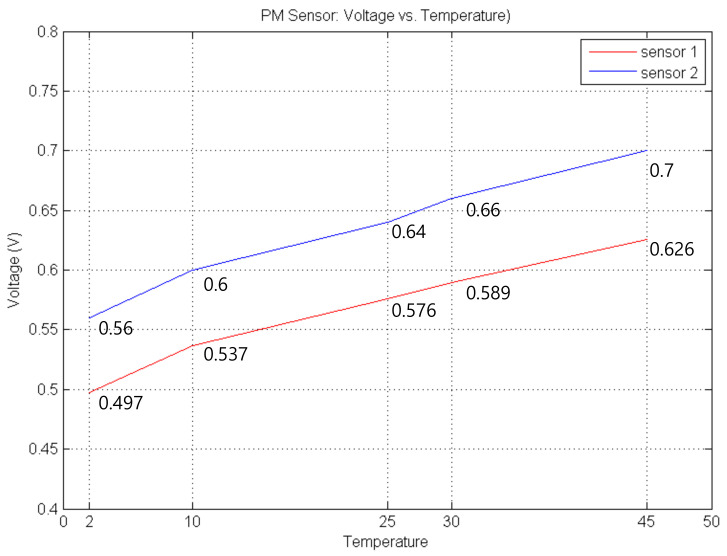
Offset according to ambient temperature.

To compensate for the temperature effect, we increased the ambient temperature to higher than 40 °C as shown Figure 9. The result shows that although there are no particles, the output voltage of the PM sensor increases up to 35 milli-voltage (6 μg/m^3^) density. To compensate for the temperature, we added offset by temperature as shown in Equation (3)

(3) $$px=a\cdot \left(x+\alpha T\cdot \Delta T\right)+b$$

where, αT is the difference between ambient temperature and the initially stored temperature in the flash memory, and ΔT is offset voltage, 3.1 milli voltage, derived empirically from Figure 10. In the Figure, the difference between 2 °C and 45 °C is 129 milli voltage, which results in approximately 23 μg/m^3^. This difference should be compensated. For example, temperature difference between summer and winter seasons is more than 40 °C. In the worst case, assume that this sensor is used in a vehicle. The temperature inside the car increases up to more than 70 °C. Therefore, the temperature compensation is necessary for better accuracy. The voltage increment is caused by Dark current of the photodiode in the PM sensor [33,34]. This leakage doubles for each 8–10 °C temperature increase.

**Figure 11:**
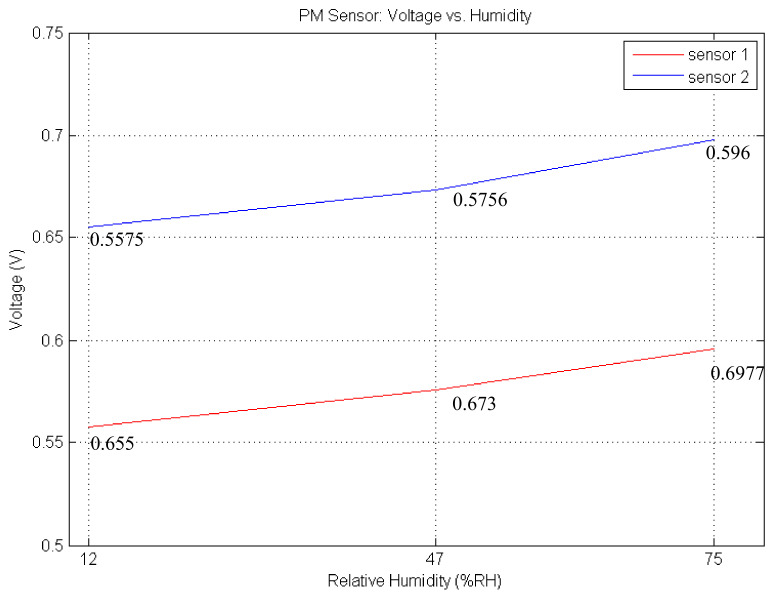
Offset according to humidity.

The last uncertainty is humidity in the particle. Therefore, we determined a relation equation between humidity and voltage when there are no particles in the chamber. We changed relative humidity from 12% to 75% RH with a temperature of 27 °C. To measure humidity and temperature, we used Silicon labs SI7020 sensors. As shown in Figure 11, the curve of Sensor 2 shows that the difference between 12% and 75% is 38 milli-voltage (approximately 6.5 μg/m^3^) and gradient is approximately 0.0006. This difference is compensated by Equation (4).

(4) $$p(x)=a\cdot \left(x+\alpha T\cdot \Delta T+\beta H\cdot \Delta H\right)+b$$

where, βH is the difference between current relative humidity and the initially stored relative humidity in the flash memory, and ΔH is offset voltage, 0.64 milli voltage, derived by the average of sensors in Figure 11, which can result in an error of approximately 0.1 μg/m^3^ per 1%RH.

To compensate for the errors by temperature and humidity, we added temperature and humidity information during the initial calibration phase as shown in Figure 7a.

### 4.2. Smartphone Application

**Figure 12:**
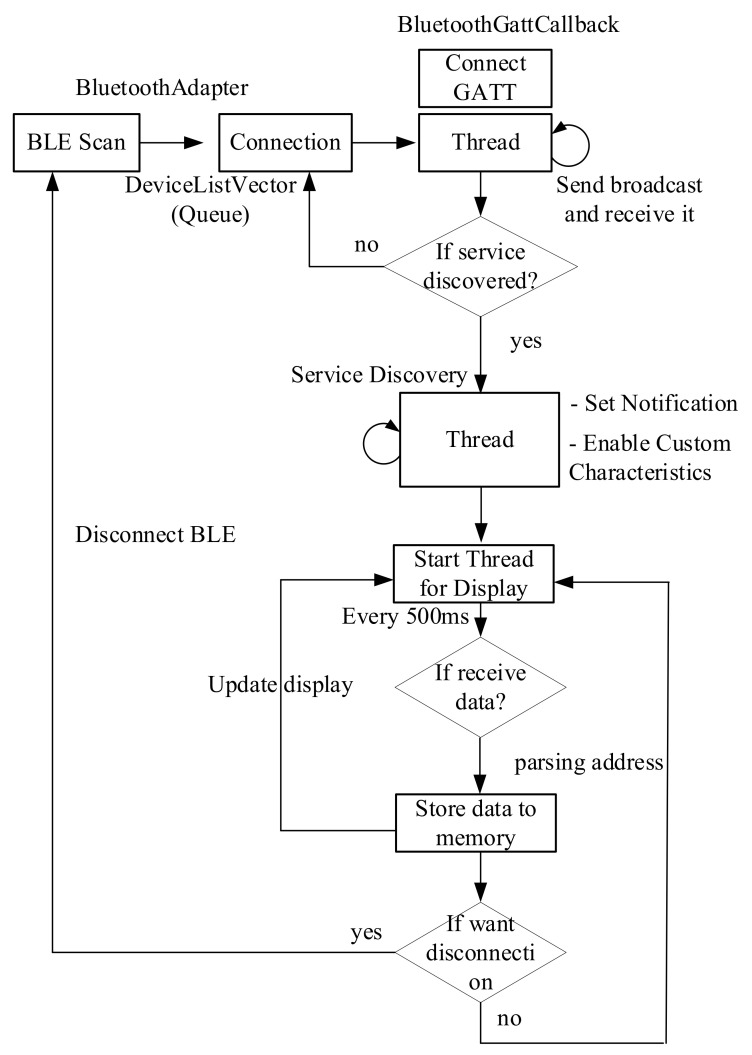
Android architecture for the PM sensing system.

The data corrected through the PM sensing system is transmitted to the smartphone through Bluetooth low energy. Figure 12 shows the operation process of the Android app for data collection. The smartphone app is designed to acquire data by communicating with seven PM sensing systems at the same time. First, when the Android app is started, it searches through the device names of the PM sensing systems that exist within the communication range, and displays the search results on the screen as a list. When the user selects PM sensing systems from which to collect data, the Android app stores the MAC addresses of the selected PM sensing systems in a queue and establishes a connection.

After that, the application calls the BluetoothGattCallback class and goes through a series of connection, service discovery, and read/write/notification processes. A delay can occur because this series of processes must proceed through all the PM sensors. To minimize this delay, the Android app allows all PM sensors to operate independently through Thread.

After a successful service discovery, the application can now start reading, writing, and enabling notifications or indications, etc. To connect multiple devices, all devices report that their services have been discovered, then BluetoothGattCallback will have all incoming data from the connected PM sensing systems. The application transfers a broadcast with the received data to the internal parser. We implemented an embedded parser to handle the received data. The analyzed data is converted into a certain format and stored in the internal file system, and displayed on the GUI along with ID information.

## 5. Performance Evaluation

**Figure 13:**
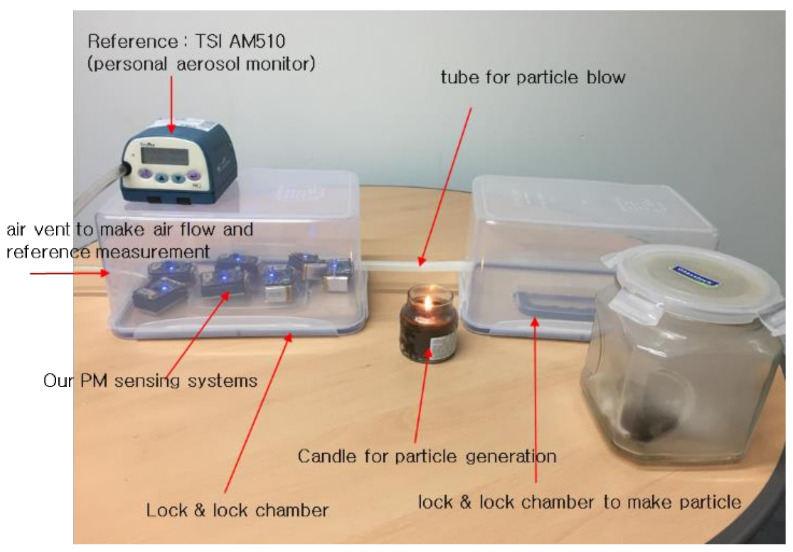
Experimental environment.

Figure 13 shows an experimental environment that consists of reference instrument TSI AM510 [35], test chambers, and the proposed systems. The reference instrument, TSI AM510, is an aerosol monitor used for the reference instrument. The chamber system is separated into two: one is to make and aggregate particles, and the other is used to measure density of particles. The two chambers are connected by a tube used to blow air. We used candle and smoke particles for our evaluation.

**Figure 14:**
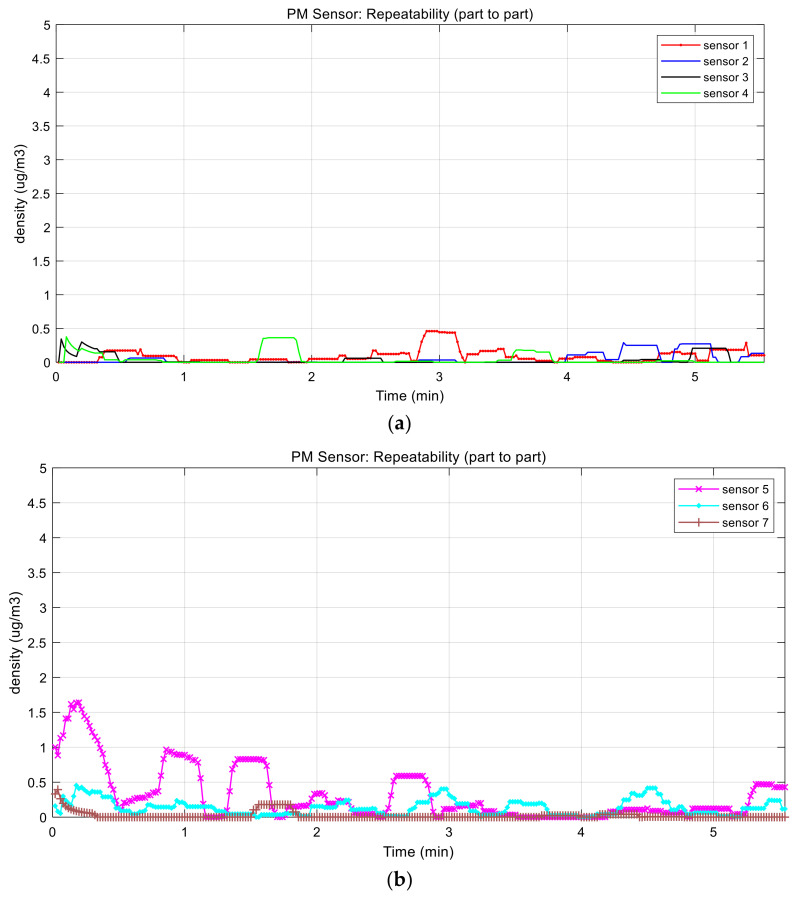
Performance evaluation: part-to-part repeatability: (**a**) data collected from sensor 1 to 4 and (**b**) data collected from sensor 5 to 7.

First, we evaluated the part-to-part repeatability of commercial PM sensors, making the system inaccurate, with respect to the different baseline voltage. Specifically, sensor 7 has the highest baseline voltage and sensor 5 has the lowest baseline voltage, as shown in Figure 7, even though there are no particles in the chamber. When converting them into concentration, the two sensors had a 70 μg/m^3^ difference. After adopting the proposed baseline voltage calibration, the result shows just less than 2 μg/m^3^ as shown in Figure 14, Table 2 and Table 3.

**Figure 15:**
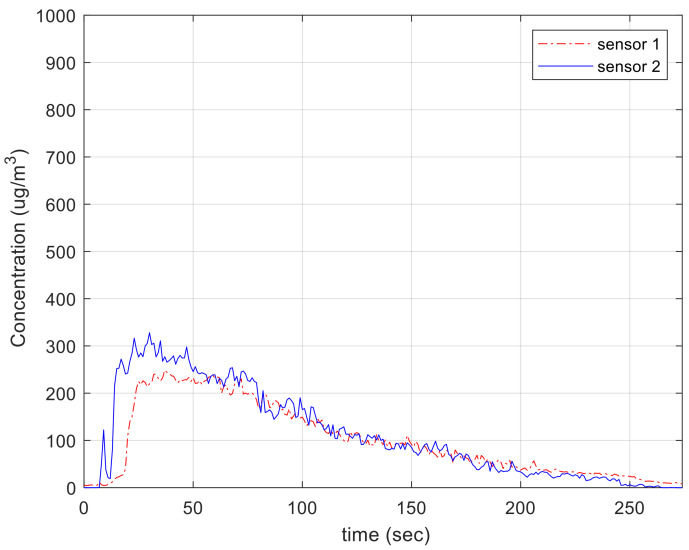
Performance evaluation: part-to-part repeatability using actual particles.

Then we conducted the performance evaluation of the two sensors (sensor 1 and sensor 2) including the proposed method with generated particles in the chambers. The evaluation was performed in room temperature (32 °C) with a relative humidity of 61%, and particle was produced by the candle as shown in Figure 13. It was too hard to generate the exact concentration of particles continuously and uniformly. Therefore, we just monitored the tendency of the two sensors as illustrated in Figure 15. Error between two sensors, as calculated by Equation (5), was approximately 10%. At the time of injecting dust particles, there was variation between the sensors because the dust was not evenly spread in the chamber. After particles were well distributed in the chamber, the error between the sensors decreased.

(5) $$\mathrm{error}=\frac{\left|C_{S1}\left(t\right)-C_{S2}\left(t\right)\right|}{C_{S1}\left(t\right)}\times 100$$

**Figure 16:**
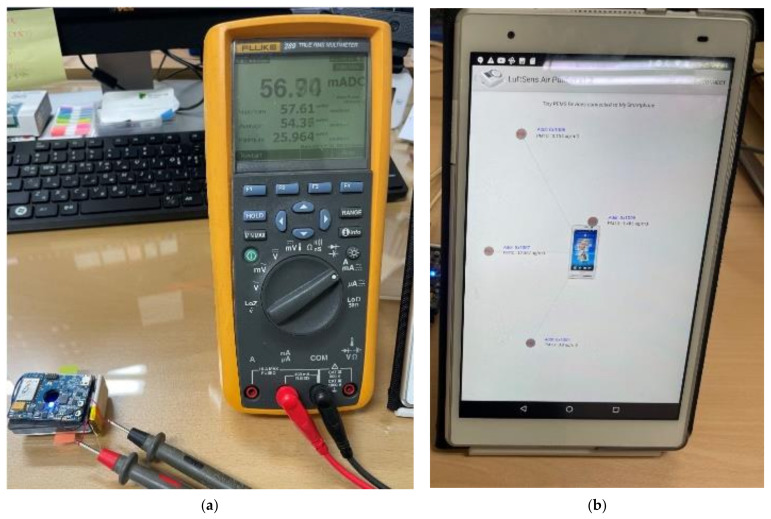
Power consumption and GUI of the smartphone: (**a**) environment for power consumption measurement (**b**) Smartphone GUI.

Then, we measured the power consumption of the system because it could be used for portable systems. Figure 16 shows the picture of the evaluation and Table 4 shows the power consumption of the system.

The system consumes approximately 57 mAh at 3 V operating voltage, i.e., 171 mW. The PM sensor uses 33.3 mA and the Bluetooth module consumes 20.7 mA at 3 V operating voltage. Other components such as MCU, temperature/humidity sensor, battery gauge, etc., use 3 mA current. Assuming that the system contains 240 mAh battery and works continuously, it can run up to 4.2 h. As a result, the lifespan of the system depends on the capacity of the battery.

**Figure 17:**
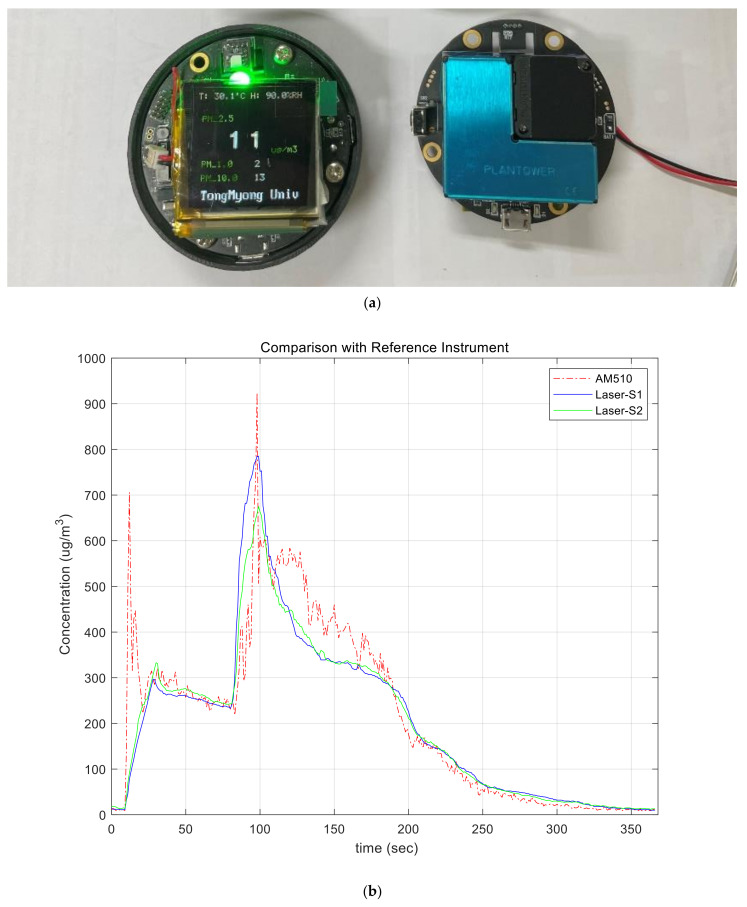
Laser-scattering-based sensor system and evaluation: (**a**) prototypes and (**b**) evaluation result.

Finally, we applied the method proposed to a laser sensor to verify that the method works correctly even when applied to other commercial sensors. Figure 17a shows the sensor and measurement system additionally fabricated to acquire the data from the actual laser-scattering-based sensor. The same MCU, NXP MKL17Z256, was used to run the proposed method, and Plantower PMS A003 [36] was used to compare the performance with the reference instrument. It is necessary to evaluate the performance by comparing it with the ground-truth value, but it is difficult to get it because of many reasons such as convection, different sizes of particles, etc. What is used as a ground-truth value is a device based on the gravimetric method worth hundreds of millions of dollars, and it is currently used as a national measurement system and platform in many countries. Instead, the evaluation to prove the proposed method was conducted by comparing TSI’s aerosol monitor, TSI AM510, as a reference instrument. This TSI AM510 device is shown to have an error within 10% of the gravimetric value in the data sheet. OLED display was additionally mounted on the board equipped with the laser PM sensor to show the measurement result, and the measured data was transmitted to the PC via USB interface. The PM sensor used in this system does not generate the analog data, but outputs the measured and corrected results as digital data in the concentrations of PM1.0, PM2.5, and PM10, respectively, through the lightweight processor inside the PM sensor. The result value is transmitted to the MCU through the UART interface, and the MCU does not need to convert the analog data to the concentration of particulates, and the measurement noise called as the short-term error is removed through the sensing frame in Figure 5. In addition, the part-to-part variation of the PMS A003 sensor based on the data sheet is an error of ±10% at a concentration of 100 to 500 μg/m^3^ and an error of ±10 μg/m^3^ at a concentration below 100 μg/m^3^ [36]. That is, the deviation between the two sensors can occur up to 20 μg/m^3^ at 100 μg/m^3^ and up to 100 μg/m^3^ at 500 μg/m^3^. This may ultimately affect the variation between systems. We also used the same baseline fitting method shown in Figure 7 for the initial calibration process to remove the deviation between the PM sensors, but the concentration value of particulate matters was directly used as it is, not the voltage value.

Another characteristic of the laser-based PM sensor is that it has no effect on temperature unlike the previously used low-cost sensor, which seems to have corrected the temperature deviation in the internal lightweight processor of the PM sensor. The reason is that a photodiode is also used in the laser PM sensor to receive the amount of light, and dark current caused by the difference of the temperature of the photodiode is unavoidable [33]. We also used the same temperature sensor, SI7020, for the temperature and humidity sensor. In the case of the experiment using this laser sensor, the effect on temperature was excluded and only correction for humidity was applied. For the experiment, AM510 equipment and two laser sensor-based measuring devices were put into the chamber to measure the concentration of particles, and the results measured over time were examined. The temperature was 29 degrees and the humidity was 55.8% RH.

Figure 17b shows a graph showing the measurement results of the reference instrument AM510 and two laser sensors. As a result of the experiment, the error between AM510 and sensor 1 was about 4.4%, the error between AM510 and sensor 2 was about 6.1%, and the error between sensor 1 and sensor 2 was about 1.8%, indicating that variation between sensors was significantly reduced. Equation (5) was used to obtain the error. It turned out that the measured error was significantly lower than 20% of first approval of the particulate matter certification standard. In addition, the difference between the reference instrument and the sensors was significantly decreased under the concentration of 300 μg/m^3^. Actually, this laser-scattering-based PM sensor manufacturer also recommends the practical measurement range as 0~500 μg/m^3^ even if it can measure over 500 μg/m^3^. In fact, WHO recommends refraining from going out when there is more than 50 μg/m^3^ of particulate matters in the air and it is hard to exceed 200 μg/m^3^ in many countries except some from Asia. In Figure 17b, it seems that the error of the reference instrument is higher than that of the sensors equipped with the proposed methods. In the case of the reference instrument, it uses only information sensed every second and does not adopt the time-weighting filter such as a low pass filter. As a result, its output signal fluctuates. Although the number of devices is small, it was confirmed that there was a variation of 2% or less, which is five times less than the 10% of the variation between the sensor described in the manufacturer datasheet.

To prove the superiority of the proposed system, its performance should be compared to that of the existing system while varying dust concentration. However, we did not carry out the comparison because it is nearly impossible to fairly spread the exact concentration of the dust in the chamber. Spreading dusts through convection is difficult and the dust sinks over time. We will perform this evaluation with a specialized institute that has special instrument for evaluation in the near future.

## 6. Conclusions

These days, air pollution, which includes dangerous materials like nitrogen, carbon, particulates, toxic gas, etc., is a global problem. Specifically, particulate matters cause damage to lung tissue and cause lung diseases such as asthma, cancer, etc.

Using the gravimetric method is an international standard, and there are several other measurement methods like beta ray, micro-balancing, and light scattering. It is common to use light scattering based on IR LED and Laser for IoT devices and electronic targets such as household air purifiers and handheld air quality monitoring systems. However, small particulate matters sensors based on light scattering are vulnerable to ambient environment. This paper described the error factors of low-cost PM sensors, i.e., short-term noise, part-to-part variation, and environmental noise, and how to correct them. The sensing frame and subframe were used to correct short-term noise, and baseline calibration was performed to correct part-to-part variation. To correct the effect by temperature and humidity that reduced the accuracy of the sensor, correction equation was added. Therefore, this study proposed correction methods for the error factors caused by the optical scattering method. We also designed and implemented the hardware and software to realize our approach. We expect the system and methods to be used as the basis for various IoT devices and air pollution measurement systems. However, since the sensor used in this study is based on IR LED, the intensity of light is weak. Compared to laser sensors with strong light intensity, the proposed system cannot classify the size of particles. However, if the calibration method proposed in this study is used for laser sensors, the performance improvement effect can be noticed in the laser-based PM sensor.

The future work includes an evaluation using the real dusts in a special chamber to prove the superiority of the proposed system and methods.

## Figures and Tables

**Table 1:** Part list used in the system.

# Part	Name	Description
1	MCU	NXP MKL17Z256VMP4 (Cortex-M0+)
2	PM sensor	Sharp GP2Y1010
3	Battery Gauge	Maxim MAX17058
4	Battery charger	TI BQ24210
5	RS-232	Silicon Labs CP 201x USB-to-RS232 bridge
6	Step-up DC-DC converter	Maxim MAX8815A
7	Bluetooth LE	Microchips RN4020
8	OP AMP	Analog Device ADA4505
9	LDO	STM STLQ015XG30R
10	Humidity/Temperature Sensor	Silicon Labs SI7020

**Table 2:** Evaluation result of the low-cost sensor in the air.

Original PM Sensor with No Calibration							
PM	#1	#2	#3	#4	#5	#6	#7
MIN	0	0.001	0.001	0.001	0.001	0.001	0.006
MAX	101.022	77.553	72.281	45.750	64.628	29.253	57.830
STD	12.913	13.838	11.170	5.213	10.088	3.875	7.355
AVG	17.844	17.290	15.812	15.191	14.578	13.230	21.283

**Table 3:** Evaluation result of the proposed system in the air.

Our Method with Calibration							
PM	#1	#2	#3	#4	#5	#6	#7
MIN	0	0	0	0	0	0	0
MAX	0.461	0.291	0.345	0.377	1.641	0.454	0.394
STD	0.101	0.082	0.068	0.093	0.378	0.110	0.055
AVG	0.093	0.046	0.032	0.045	0.309	0.136	0.021

**Table 4:** Power consumption of the system.

	PM Sensor	Bluetooth	Others (MCU, H/T, Etc.)	Total
Consumed current (mA)	33.3 mA @3 V	20.7 mA @3 V	3 mA @3 V	57 mA

## Data Availability

Not applicable.
